# Impact of ERAS compliance on the delay between surgery and adjuvant chemotherapy in hepatobiliary and pancreatic malignancies

**DOI:** 10.1007/s00423-020-01981-1

**Published:** 2020-09-11

**Authors:** Pénélope St-Amour, Pascal St-Amour, Gaëtan-Romain Joliat, Aude Eckert, Ismail Labgaa, Didier Roulin, Nicolas Demartines, Emmanuel Melloul

**Affiliations:** 1 Department of Visceral Surgery, Lausanne University Hospital (CHUV), Rue du Bugnon 46, 1011 Lausanne, Switzerland; 2 Department of Economics, HEC Lausanne, University of Lausanne (UNIL), Lausanne, Switzerland; 3 Department of Biology and Medicine, University of Lausanne (UNIL), Lausanne, Switzerland

**Keywords:** ERAS, Liver surgery, Pancreatic surgery, Adjuvant chemotherapy

## Abstract

**Background:**

Multidisciplinary approach with adjuvant chemotherapy is the key element to provide optimal outcomes in pancreas and liver malignancies. However, post-operative complications may increase the interval between surgery and chemotherapy with negative oncologic effects.

**Hypothesis and study aim:**

The aim of the study was to analyse whether compliance to Enhanced Recovery After Surgery (ERAS) pathway was associated with decreased interval to adjuvant chemotherapy.

**Methods:**

Retrospective analysis of all consecutive ERAS patients with surgery for hepatobiliary or pancreatic malignancies at the University Hospital of Lausanne between 2012 and 2016. Multivariate analysis was performed to assess the impact of ERAS compliance on time to chemotherapy.

**Results:**

A total of 133 patients with adjuvant chemotherapy were included (*n* = 44 liver and *n* = 89 pancreatic cancer). Median compliance to ERAS was 61% (IQR 55–67) for the study population, and median delay to chemotherapy was 49 days (IQR 39-61). Overall, compliance ≥ 67% to ERAS induced a significant reduction in the interval between surgery and chemotherapy for young patients (< 65 years old) with or without severe comorbidities (reduction of 22 and 10 days, respectively). High compliance in young ASA3 patients with liver colorectal metastases was associated with an increase of 481 days of DFS.

**Conclusions:**

ERAS compliance ≥ 67% tends to be associated with a reduction in the delay to adjuvant chemotherapy for young patients with hepatobiliary and pancreatic malignancies. More prospective studies with strict adhesion to the ERAS protocol are needed to confirm these results.

## Introduction

Pancreas and liver malignancies are common tumours with increasing incidence and poor prognosis. Five-year survival is 9% for all stages of pancreas malignancies and 18% for liver malignancies [1]. To improve this unfavourable outcome, multidisciplinary approach including neo- or adjuvant chemotherapy combined with surgical resection has become the best treatment to offer long-term disease-free (DFS) and overall survival (OS).

The ESPAC-3 trial showed that the most important factor for long-term survival in pancreas adenocarcinoma was to achieve complete cycles of adjuvant chemotherapy. However, if it is not possible to undergo full cycles, the recommended strategy is to wait for a full post-operative recovery rather than to start early without being able to finish the treatment [2]. On the other hand, delayed chemotherapy after 12 weeks would not be associated with a decreased long-term survival for pancreatic cancer, because adjuvant chemotherapy is a prognostic factor by itself, so it should be offered even later [3]. For colorectal cancer and its liver metastases, it is well demonstrated that adjuvant chemotherapy should be initiated within 6 to 8 post-operative weeks to offer the best long-term outcome [4–6]. Therefore, the timing for adjuvant chemotherapy is one of the key elements in patients with hepatobiliary and pancreatic malignancies. For this reason, recovery after surgery should be optimal to allow chemotherapy beginning as soon as the patients are fit enough.

Enhanced Recovery After Surgery (ERAS) pathway has been developed to reduce the negative effect of the surgical stress. Numerous meta-analyses have shown the benefits of an ERAS pathway implementation in pancreas and liver surgery [7–11]. The principal advantages are a reduction in medical complications after surgery, reduced length of stay and decreased costs. Guidelines for ERAS protocol are based on these studies and applied in many hospitals worldwide [12–14]. Achieving the same goals, recent studies have shown the benefits of introducing other similar pathways, especially targeting perioperative rehabilitation and more precisely pre-habilitation [15].

The question remains whether ERAS pathways have an impact on the time to receipt of adjuvant chemotherapy. Moreover, data on the association between the compliance to ERAS and the increase of DFS and OS are scarce.

The aim of this study was to analyse the impact of ERAS compliance on the delay to adjuvant chemotherapy in patients operated for liver and pancreas malignancies.

## Material and methods

### Data

This is a retrospective analysis of a prospectively maintained database. All included patients underwent pancreatic or liver surgery for malignancies between 2012 and 2016, and all were included in an ERAS protocol [12, 13]. In addition, they all were discussed in multidisciplinary tumour board (MDT) meeting and had an indication to receive adjuvant chemotherapy after surgery. Tumours included were pancreas ductal adenocarcinoma, cholangiocarcinoma, liver colorectal metastasis and gallbladder adenocarcinoma. Patients with liver colorectal metastases who benefitted from a reverse treatment without chemotherapy between the hepatic and colorectal surgery were excluded from the analysis (*n* = 15). Pre-operative patients’ characteristics such as age, gender, BMI, smoking and alcohol drinking habits and ASA score (American Society of Anesthesiology) were included as cofactors. Post-operative data, such as CCI score (Comprehensive Complication Index), were also used as explanatory variables. Following standard practices, patients who died within 90 days after surgery were excluded of the survival analysis, since their death was more likely linked to the complications of surgery than to the oncological disease [16–18].

Adjuvant chemotherapy was defined as systemic oncologic treatment (chemotherapy) initiated within 200 days after surgery as previously defined [3, 19]. If chemotherapy was initiated after a recurrence of the disease, it was considered as a palliative treatment and thus not considered as adjuvant. These cases were excluded from the analysis (*n* = 10). Total compliance to ERAS pathway was calculated by a software program developed by ERAS society to provide a numerical score to compliance, taking into account pre-admission and pre-, intra- and post-operative compliances (EIAS: ERAS Interactive Audit System). Measurement of these compliances is principally based on data concerning the patients’ hospitalisation (i.e. post-operative early refeeding and mobilisation, weight’s evolution), collected by nurses and medical assistance personnel. Data concerning patients’ disease and perioperative information (i.e. nausea prevention, surgical duration) are also collected by the anaesthetist and the surgeon. The patient is himself strongly involved, during all the process. All this information is then summarised by an ERAS dedicated clinical nurse in a form on the software, and the percentage of compliances is generated. This study was approved by the local Ethics’ Commission (Project-ID 2019-01097).

### Outcomes analysed

The primary outcome analysed was the delay (measured in days) between the date of surgery and initiation of the first cycle of adjuvant chemotherapy.

### Statistical analyses

Multivariate regression analysis (using STATA© software) was used to study the effects of high compliance with ERAS guidelines on the time to receipt of adjuvant chemotherapy. More precisely, we resorted to a maximum likelihood estimation of a fully parameterised survival time (ST) model for the time to adjuvant chemotherapy, assuming a Weibull distribution. The uncensored sample was restricted to the observations with achieved outcome (e.g. 133 out of 173 cases received adjuvant chemotherapy). For robustness and comparison with the literature, this multivariate procedure was also applied for two other outcomes of interest, time to recurrence and time to death in order to analyse DFS and OS [2, 3, 20].

In order to control for patient-specific characteristics, clinically relevant covariates were included such as gender, as well as health-related habits (smoking, alcohol drinking, high BMI and CCI as well as ASA score), in the time to outcome analysis. To facilitate the interpretation of the analysis, binary variables relying on the 75th percentile for BMI, ERAS compliance and CCI were generated (describing the higher part of the distribution). The main regressor of interest was high compliance to ERAS and was thus considered as a compliance of ≥ 67% (75th percentile). It was interacted with an old age indicator (age > 65 years old), as it is known that age is related to worse survival, especially after pancreatic cancer surgery [21, 22]. The use of interaction terms allowed calculating the effects of high compliance for the entire data sample, as well as for sub-groups stratified by age. We also considered a triple interaction of compliance, age and ASA in additional testing, and all our main results were robust to this alternative specification.

In the attempt to compare our results with available literature, we also tested the same analyses with a threshold of a hundred days for initiation of adjuvant chemotherapy [2, 23], and the results were qualitatively similar to those mentioned here.

Moreover, in order to control for the type of illness heterogeneity, we performed two types of analyses. First, the type of malignancy (liver versus pancreas) was included as a regressor interacted with compliance to differentiate the effect of compliance by type of cancer. Our results were qualitatively similar. Second, we also analysed heterogeneity by separate regressions by type of cancer subgroups (liver colorectal metastases versus pancreas adenocarcinomas). The associated results are discussed below.

The reported regression output refers to the marginal effect of high compliance, measured in changes in median time to outcome.

## Results

### Descriptive statistics

**Fig. 1:**
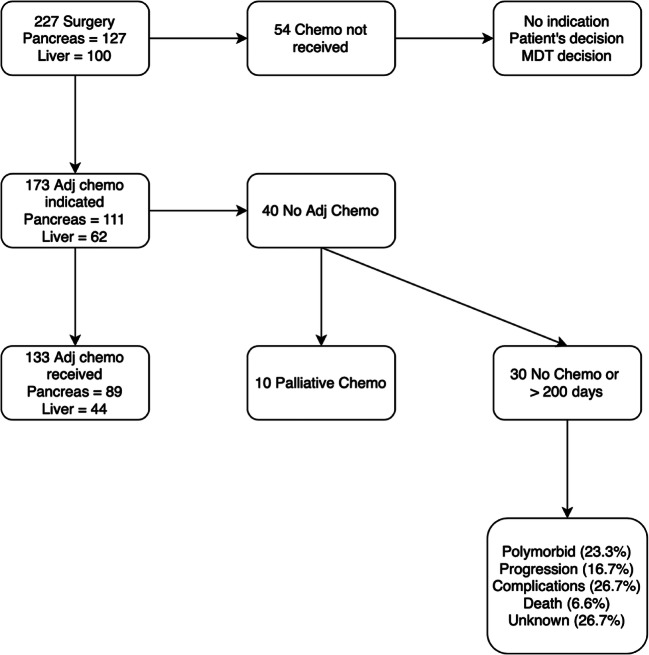
Flowchart. Description of patients’ inclusion in the study group for analysis. Adj adjuvant, Chemo chemotherapy, MDT multidisciplinary tumour board meeting

Figure 1 describes the study flow chart. Among patients included in the ERAS database, 227 had surgery for pancreatic or liver malignancies defined by the inclusion criteria between 2012 and 2016 (*n* pancreas = 127; *n* liver = 100). Among them, 173 had an indication to adjuvant chemotherapy after MDT discussion. Finally, 40 patients (23%) did not receive the adjuvant treatment within 200 days after surgery because of comorbidities (*n* = 7), progression of the disease after post-operative imaging reassessment (*n* = 15), complications (*n* = 8) or early death (*n* = 2) (unknown reason in 8 cases). These patients had a median compliance to ERAS of 59% (IQR 52–64%). The main study group therefore included 133 patients who received adjuvant chemotherapy, at a known date (*n* pancreas = 89; *n* liver = 44). In the pancreas group, all the patients had adenocarcinoma. In the liver group, 37 patients had colorectal metastases, one patient had intrahepatic cholangiocarcinoma, five patients had hilar cholangiocarcinoma and one patient had gallbladder adenocarcinoma. Concerning the liver colorectal metastases, the majority (*n* = 30, 81%) had synchronous lesions. Only one fourth (*n* = 9, 24%) benefitted from a reverse treatment, and only 2 patients (5%) had a one-stage procedure. Median compliance to ERAS for patients with liver malignancies was 100% pre-admission (IQR 58–100%), 86% pre-operative (IQR 86–88%), 67% intra-operative (IQR 57–71%) and 28% post-operative (IQR 15–46%). For pancreas malignancies, it was 100% pre-admission (IQR 67–100%), 100% pre-operative (IQR 100–100%), 100% intra-operative (IQR 75–100%) and 36% post-operative (IQR 20–46%). Due to the low number of cases in each group, more analysis could not be performed. Descriptive characteristics of the patients are summarised in Table 1.

**Table 1:** Patients’ characteristics

Variables	Liver *n* = 62 (36%)	Pancreas *n* = 111 (64%)	All *n* = 173 (100%)
Female	23 (37%)	60 (54%)	83 (48%)
Smoking	23 (37%)	32 (29%)	55 (32%)
Alcohol	8 (13%)	12 (11%)	20 (12%)
ASA 3	17 (27%)	43 (39%)	60 (35%)
Age (years)	63 (IQR 55–69)	68 (IQR 61–73)	67 (IQR 58–72)
BMI (kg/m^2^)	25 (IQR 23–28)	25 (IQR 23–28)	25 (IQR 23–28)
ERAS compliance (%)	54 (IQR 48–61)	63 (IQR 58–70)	61 (IQR 55–67)
CCI	0 (IQR 0–30)	23 (IQR 12–39)	21 (IQR 0–35)

Patients’ demographic characteristics for all the study population and by subgroup of liver or pancreas malignancies

*ASA* American Society of Anesthesiologists, *BMI* body mass index, *ERAS* Enhanced Recovery After Surgery, *CCI* Comprehensive Complication Index

For all the study population who received adjuvant chemotherapy (*n* = 133), the median time between surgery and receipt of adjuvant chemotherapy was 49 days (IQR 39–61). Median DFS was 13 months (IQR 5–22), and OS was 34 months (IQR 25–42) for the patients with liver colorectal metastases. Patients with pancreas malignancies had a median DFS of 12 months (IQR 8-22) and median OS of 20 months (IQR 13-36).

Compliance to ERAS was comparable (*p* = 0.25) in patients < 65 years old (median compliance 61%) and > 65 years old (median 62%). Moreover, it was also comparable regarding the ASA score (median compliance 61% for ASA1–2 and ASA3, *p* = 0.35) and the CCI score (median compliance 58% for high CCI, 61% for low CCI, *p* = 0.10).

## Multivariate analyses

### Time to adjuvant chemotherapy

#### Overall population

**Fig. 2:**
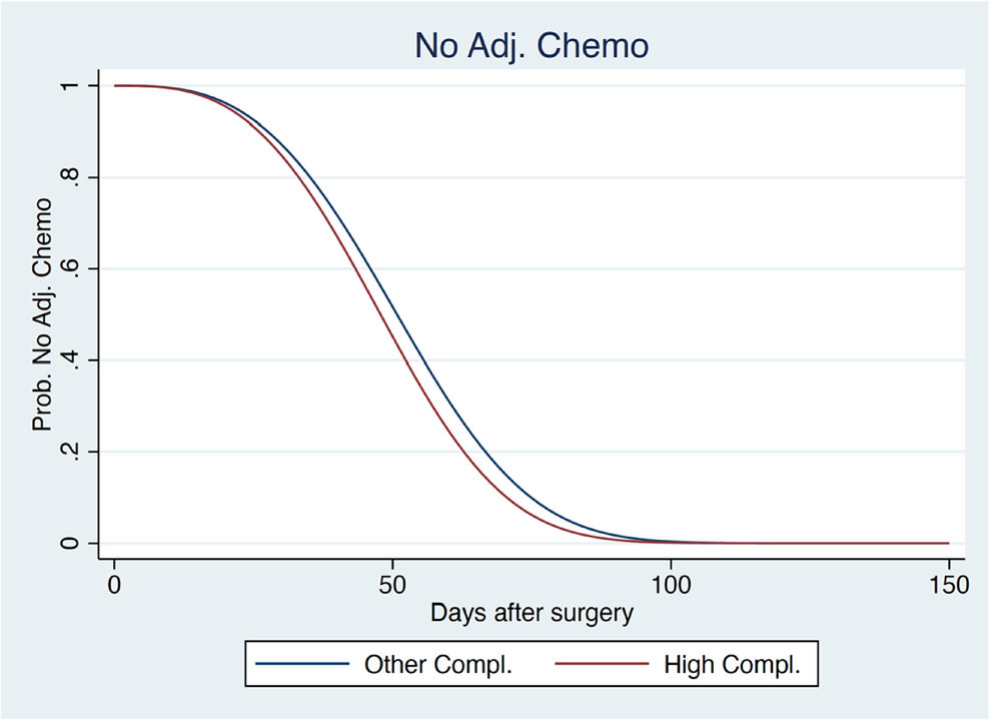
Probability no adjuvant chemotherapy for all study population. Weibull distribution of the probability not to have an adjuvant chemotherapy at each moment after surgery in days, for high compliers (compliance ≥ 67%) versus other compliances, for all patients with hepatobiliary and pancreas malignancies. Note that for this graph, *n* Low Compl. = 91 and *n* High Comp. = 42, *p* = 0.37. Adj chemo adjuvant chemotherapy, Compl compliance, Prob probability

Table 2 displays the marginal effects (measured in changes in median waiting time) of the explanatory variables on the time to receipt of adjuvant chemotherapy for all the study population (i.e. exogenous variables not affected by our outcomes variables). Compliance > 67% to ERAS had no effect on the time to receipt of adjuvant chemotherapy when considering the whole study population (Fig. 2).

**Table 2:** Time to chemotherapy (all patients)

Variables	Chg. med. time	Std. err.	*z*	*P* > *z*	95% conf. interval	
Female	6.209	2.983	2.080	0.037	0.361	12.056
High BMI	− 1.539	3.211	− 0.480	0.632	− 7.832	4.754
Smoker	− 6.346	3.028	− 2.100	0.036	− 12.280	− 0.412
Alcohol drinking	12.121	4.122	2.940	0.003	4.043	20.199
High CCI	7.820	3.554	2.200	0.028	0.854	14.786
High compliance	− 4.490	3.018	− 1.490	0.137	− 10.404	1.425
Age > 65	0.412	3.518	0.120	0.907	− 6.483	7.306
ASA3	13.646	5.120	2.670	0.008	3.611	23.681

Multivariate analysis of time to chemotherapy for all the study population

*BMI* body mass index, *CCI* Comprehensive Complication Index, *ASA* American Society of Anesthesiologists, *Chg. Med. Time* change in median time, *Std. Err* standard error, *95% Conf. Interval* 95% confidence interval

**Fig. 3:**
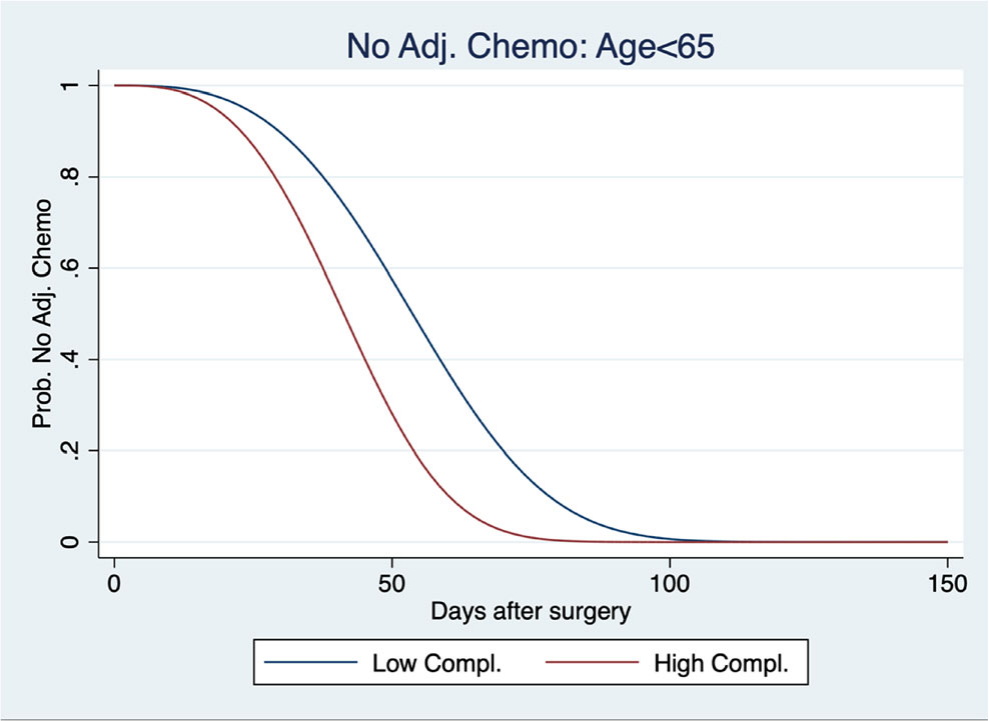
Probability no adjuvant chemotherapy for young patients. Weibull distribution of the probability not to have an adjuvant chemotherapy at each moment after surgery in days, for high compliers (compliance ≥ 67%) versus other compliances and for young patients (< 65y.o.) with hepatobiliary and pancreas malignancies. Note that for this graph, *n* Low Compl. = 40 and *n* High Comp. = 15, *p* = 0.001. Adj chemo adjuvant chemotherapy, Compl compliance, Prob probability

When stratified by age, the subgroup analysis showed that compliance > 67% to ERAS reduced the interval to chemotherapy of 12 days for younger patients (< 65 years old, *p* = 0.001) (Table 3). The effect on shorter waiting time is illustrated in Fig. 3, which plots the probability of no adjuvant chemotherapy, in function of days after surgery, with the compliance to ERAS in the subgroup of young patients. Consistent with the regression analysis, at each period after surgery, young high compliers were significantly more likely to benefit from adjuvant chemotherapy. For robustness, we also performed the analyses with interactions of ASA score and age. Those analyses showed that young ASA1–2 patients had a reduction in the delay of 10 days (*p* = 0.002) and ASA3 patients of 22 days (*p* = 0.021), indicating stronger effects in the higher ASA subgroup.

**Table 3:** Time to chemotherapy—by age subgroup multivariate analysis (all patients)

Age (years)	Chg. Med. time	Std. err.	*z*	*P* > *z*	95% conf. interval	
< 65	− 12.089	3.673	− 3.290	0.001	− 19.287	− 4.891
≥ 65	0.869	4.121	0.210	0.833	− 7.207	8.946

Multivariate analysis of time to chemotherapy for all the study population, interacted with age

*Chg. Med. Time* change in median time, *Std. Err* standard error, *95% Conf. Interval* 95% confidence interval

#### Subgroup analyses—liver malignancies

Liver colorectal metastases were analysed separately. A reduction of 14 days in the delay to adjuvant chemotherapy was found in young high compliers (*p* = 0.043) (Table 4).

**Table 4:** Time to chemotherapy—by age subgroup multivariate analysis (liver colorectal metastases)

Age (years)	Chg. Med. time	Std. err.	*z*	*P* > *z*	95% conf. interval	
< 65	− 13.492	6.677	− 2.020	0.043	− 26.578	− 0.405
≥ 65	7.491	7.882	0.950	0.342	− 7.957	22.939

Multivariate analysis of time to chemotherapy for patients with liver colorectal metastases, interacted with age

*Chg. Med. Time* change in median time, *Std. Err* standard error, *95% Conf. Interval* 95% confidence interval

#### Subgroup analyses—pancreas malignancies

Separate analyses concerning pancreas malignancies could not identify a significant effect of ERAS compliance on the time to receipt of adjuvant chemotherapy.

### Time to recurrence

#### Liver malignancies

Young ASA3 patients with liver colorectal metastases and high compliance to ERAS had an improvement of disease-free survival of 481 days (*p* < 0.001) (Table 5).

**Table 5:** Time to recurrence—by ASA and age subgroup multivariate analysis (liver colorectal metastases)

ASA	Age (years)	Chg. med. time	Std. err.	*z*	*P* > *z*	95% conf. interval	
1–2	< 65	− 58.004	83.506	− 0.690	0.487	− 221.672	105.664
1–2	≥ 65	15.377	140.417	0.110	0.913	− 259.835	290.588
3	< 65	480.889	130.812	3.680	0.000	224.502	737.277
3	≥ 65	831.072	520.342	1.600	0.110	− 188.781	1850.924

Multivariate analysis of time to recurrence for patients with liver colorectal metastases, interacted with age and ASA score

*ASA* American Society of Anesthesiologists, *Chg. Med. Time* change in median time, *Std. Err* standard error, *95% Conf. Interval* 95% confidence interval

High compliance to ERAS in patients with liver colorectal metastases was not associated with a significant improvement of OS on multivariate analyses. Subgroup analysis of pancreas malignancies also did not show a significant impact of ERAS compliance on DFS and OS.

## Discussion

The results of this study confirmed the positive effect of high compliance to ERAS regarding the post-operative outcomes in patients with hepatobiliary and pancreatic malignancies. In particular, an association between high ERAS compliance and a reduction in the time to receipt of adjuvant chemotherapy was identified in the subgroup of young patients (< 65 years) whatever their comorbidities.

To our knowledge, no published study has assessed the relationship between ERAS compliance and the delay to adjuvant treatment for pancreatic and liver malignancies. It seems more likely that this delay would mainly be related to post-operative complications [19, 20, 24].

Wu et al., in a retrospective study including more than a thousand patients with pancreas ductal adenocarcinoma, found that complications after surgery were associated with a lower probability to have adjuvant oncological treatment and longer delay to adjuvant chemotherapy (median difference of 7 days). Moreover, they also found a correlation between post-operative complications and lower survival, but no clear relationship between delayed chemotherapy and decreased survival [19]. Petermann et al. had similar results, with lower survival related with post-operative complications after R1 resection of pancreatic head adenocarcinoma [25]. Kamphues et al. also found the same results in patients with pancreatic head tumours [26]. On the other hand, Murakami et al., in a retrospective study of 100 patients, concluded that post-operative complications were associated with delayed adjuvant chemotherapy for pancreatic carcinoma. They also showed evidence that early initiation of adjuvant treatment (< 20 days after surgery) was associated with a higher DFS and OS [24].

Valle et al., in a derived study of the prospective randomized trial ESPAC-3, with almost one thousand patients with pancreatic adenocarcinoma, found that delayed adjuvant chemotherapy up to 12 weeks for patients with completed cycles of treatment was not associated with lower overall survival [2]. In the study by Mirkin et al., comparing a delay to chemotherapy of more or less than 12 weeks, adjuvant chemotherapy was an independent prognostic factor in patients operated for pancreatic cancer, regardless of the time of initiation [3]. Similarly, in a retrospective study of almost nine hundred patients with pancreatic adenocarcinoma, adjuvant chemotherapy was found to be an independent prognostic factor, without any negative effect of a delayed treatment after more than 12 weeks post-operatively [20]. Finally, Nakagawa et al. also suggested that completion of adjuvant chemotherapy is a main determinant of long-term survival [27]. Therefore, results are conflicting and it is not clear, for hepatobiliary and pancreatic surgery, whether the delay to adjuvant chemotherapy is more important than the completion of all cycles, whatever the delay between surgery and the start of chemotherapy might be. However, it seems clear that it is more important to start an adjuvant treatment in a fit patient in order to complete all cycles of chemotherapy rather than starting too early in an unfit patient who would not tolerate the full cycles. The performance status of the patient with pancreatic adenocarcinoma is one of the main points in the decision to start adjuvant chemotherapy, which offers better outcomes [28].

In colorectal cancer, there are no studies analysing the effect of delayed chemotherapy on survival in patients with liver metastases. In a systematic review and meta-analysis, Biagi et al. concluded that delayed adjuvant chemotherapy in colorectal cancer, all stages confound, was associated with lower survival [4]. Kang et al. assessed the feasibility of initiating adjuvant chemotherapy before hospital discharge after surgery for stage II–IV colon cancer. They found no difference in cycle completion rates of chemotherapy for the two groups (in-hospital initiation vs outpatients). On the other hand, they found no significant difference in OS for patients with initiation of chemotherapy before hospital discharge compared with the other ones, but initiation of the treatment within 6 weeks was associated with better prognosis [5]. A systematic review and meta-analysis of outcomes in delayed adjuvant chemotherapy (> 6–8 weeks) including gastric, colorectal and pancreatic cancers highlighted that a delayed chemotherapy was associated with a decreased survival for gastric and colorectal tumours, but not clearly for pancreatic malignancies [6].

Thus, it is recommended that adjuvant chemotherapy should be proposed for pancreatic cancer when indicated, even if delayed, and that a main point of the outcome improvement is the reduction of post-operative complications. Therefore, ERAS pathways implementation, by reducing post-operative complications, is certainly a key element to improve survival outcomes. Indeed, our study identified reduced delay to chemotherapy in young healthy and non-healthy patients. This positive effect was not observed in elderly. This might be related to the initial functional status of elderly patients that preclude in some cases the proper implementation of all ERAS items. One can argue that younger and fitter patients demonstrate a higher compliance with the entirety of recommended therapy including ERAS and multimodal oncologic therapy. Vice versa, frail or incompliant patients may have a lower compliance with both ERAS and adjuvant therapy. However, our data showed that compliance to ERAS protocol was comparable in patients < 65 years old and > 65 years old. Moreover, it was also comparable regarding the ASA score and the CCI score.

Despite similar rates of high compliers between young and old patients, we found a stronger effect in young patients, whatever the severity of their comorbidities. This could be related to a greater reduction in post-operative complications and increased opportunity to access multimodal oncologic therapies. This needs to be addressed and confirmed in future trials.

Finally, we found better compliance to ERAS items in pre-admission and pre- and intra-operative than post-operative. Reasons for low compliance remain not clearly identified, but the global effect of ERAS implementation is well demonstrated, with reduced post-operative complications (principally medical ones) and length of stay. It is thought that many other unknown factors are implied in this relationship [29].

This study has several limitations inherited from its retrospective design and inclusion of patients with different types of cancer (small number of observations when analysed separately). Indeed, we analysed the marginal effect of compliance on a subgroup of young patients, interacted with ASA score to take into account pre-operative comorbidities. The effect of high compliance to ERAS remained significant to reduce the delay to adjuvant chemotherapy, when compared with low compliers. Thus, the results confirm that high compliance to ERAS protocol has a positive impact on complications and the delay to chemotherapy in a subgroup of young patients, and it will pave the way to further studies using strict ERAS protocol in liver and pancreas cancer. This will allow benchmarking and better comparisons between studies.

## Conclusion

To conclude, high compliance to ERAS in oncologic hepatobiliary and pancreatic surgery tends to be associated with a reduction in the delay to adjuvant chemotherapy, particularly in young patients < 65 years old. Moreover, young ASA3 patients with liver colorectal metastases and high compliance to ERAS had an increased DFS. Further studies are then needed to confirm these results.

## Abbreviation

ASA: American Society of Anesthesiology
CCI: Comprehensive Complication Index
DFS: Disease-free survival
EIAS: ERAS Interactive Audit System
ERAS: Enhanced Recovery After Surgery
MDT: Multidisciplinary tumour board
OS: Overall survival
